# Neurofibromatosis type 1: evaluation by chest computed tomography

**DOI:** 10.1590/0100-3984.2020.0150

**Published:** 2021

**Authors:** Sérgio Ferreira Alves Júnior, Klaus Loureiro Irion, Alessandro Severo Alves de Melo, Gustavo de Souza Portes Meirelles, Rosana Souza Rodrigues, Arthur Soares Souza Jr., Bruno Hochhegger, Gláucia Zanetti, Edson Marchiori

**Affiliations:** 1 Universidade Federal do Rio de Janeiro (UFRJ), Rio de Janeiro, RJ, Brazil.; 2 Manchester University, NIHR Biomedical Research Centre, Manchester, United Kingdom.; 3 Universidade Federal Fluminense (UFF), Niterói, RJ, Brazil.; 4 Grupo Fleury, São Paulo, SP, Brazil.; 5 Faculdade de Medicina de São José do Rio Preto (Famerp), São José do Rio Preto, SP, Brazil.; 6 Universidade Federal de Ciências da Saúde de Porto Alegre (UFCSPA), Porto Alegre, RS, Brazil.

**Keywords:** Neurofibromatoses, Cysts, Lung diseases, Tomography, X-ray computed, Neurofibromatoses, Cistos, Pneumopatias, Tomografia computadorizada

## Abstract

**Objective:**

The aim of this study was to evaluate chest computed tomography (CT) findings in patients diagnosed with neurofibromatosis type 1 (NF1).

**Material and Methods:**

This was a retrospective study in which we reviewed the chest CT scans of 14 patients diagnosed with NF1 and neurofibromatosis-associated diffuse lung disease (NF-DLD). The sample comprised eight women and six men. The median age was 55 years (range, 11-75 years). The diagnosis of NF1 was made on the basis of the diagnostic criteria established by the U.S. National Institutes of Health. The images were analyzed by two chest radiologists, who reached decisions by consensus.

**Results:**

The predominant CT finding of NF-DLD was multiple cysts, which were observed in 13 patients (92.9%), followed by emphysema, in eight (57.1%) and subpleural bullae, in six (42.9%). Other findings included subcutaneous neurofibromas, in 12 patients (85.7%), ground-glass opacities, in one (7.1%), and tracheobronchial neurofibromas, in one (7.1%). The pulmonary abnormalities were bilateral in 12 cases (85.7%). The abnormalities were predominantly in the upper lung fields in eight cases (57.1%), and their distribution was random in 11 (78.6%).

**Conclusion:**

Pulmonary cysts, emphysema, and subpleural bullae appear to be the chest CT findings that are most characteristic of NF-DLD.

## Abstract (pt)

**Objetivo:**

O objetivo deste estudo foi avaliar os achados pulmonares na tomografia computadorizada do tórax de pacientes diagnosticados com neurofibromatose tipo 1 (NF1).

**Materiais e Métodos:**

Foram revisados, retrospectivamente, os achados tomográficos de 14 pacientes com doença pulmonar difusa associada à NF1 (NF-DPD). A amostra incluiu oito mulheres e seis homens, com idade entre 11 e 75 anos (mediana de idade de 55 anos). O diagnóstico foi estabelecido com base em critérios diagnósticos predeterminados pelo National Institutes of Health dos Estados Unidos. As imagens foram analisadas de forma independente por dois radiologistas, que chegaram a um consenso.

**Resultados:**

Os achados tomográficos predominantes foram múltiplos cistos em 13 pacientes (92,9%), enfisema em oito (57,1%) e bolhas subpleurais em seis (42,9%). Achados associados incluíram neurofibromas cutâneos e subcutâneos em 12 pacientes (85,7%), opacidades em vidro fosco em um (7,1%) e neurofibromas traqueobrônquicos em um (7,1%). As anormalidades pulmonares foram bilaterais em 12 casos (85,7%). Houve predomínio nos terços superiores em oito (57,1%) pacientes e se distribuíram randomicamente pelos pulmões em 11 (78,6%).

**Conclusão:**

Os achados tomográficos pulmonares mais frequentes na NF-DPD foram os cistos pulmonares, o enfisema e as bolhas subpleurais.

## INTRODUCTION

Neurofibromatosis type 1 (NF1), also known as von Recklinghausen disease, is an autosomal dominant genetic syndrome that affects the ectoderm and mesoderm, with a variable clinical presentation, characterized by neurofibromas, café au lait spots, freckles in the axillary or inguinal region, and the pigmented iris hamartomas known as Lisch nodules^**(1-3)**^. Severe manifestations, which are less common, include plexiform neurofibromas, malignant tumors of the peripheral nerve sheath, optic nerve gliomas, central nervous system gliomas, scoliosis, tibial dysplasia, and vasculopathy^**(1,4,5)**^. It is a relatively common disease, with an incidence of approximately 1 in 3,000 live births, 30-50% of the affected patients having no family history of the disease. The latter cases probably arise from mutations in germ cells, typically in paternal germ cells^**(1)**^.

In the chest, NF1 has a variety of manifestations, including intrathoracic neurogenic tumors, meningoceles, kyphoscoliosis, rib deformities, cutaneous/subcutaneous neurofibromas of the chest wall, bullous lung disease, and interstitial lung disease^**(1,6-8)**^. Although cases of neurofibromatosis-associated diffuse lung disease (NF-DLD) have been reported, little is known about its severity, overall prevalence, and clinical characteristics^**(6,9,10)**^.

The objective of this study was to evaluate the most common chest computed tomography (CT) findings in patients diagnosed with NF-DLD, as well as to analyze the morphological characteristics and distribution of lesions in the lung parenchyma. In addition, we investigate some epidemiological aspects of the disease, such as its distribution by patient sex and age.

## MATERIALS AND METHODS

This was a retrospective, cross-sectional, observational study of the chest CT scans of 14 patients diagnosed with NF-DLD. The patients were selected by searching the electronic medical records on file at the Hospital Universitário Clementino Fraga Filho of the Federal University of Rio de Janeiro, as well as by personal contact with radiologists at various institutions throughout Brazil.

The 14 patients in the study sample were diagnosed with NF1 on the basis of their clinical history and the criteria established by the U.S. National Institutes of Health^**(11)**^. In addition, one of the patients also underwent molecular genetic testing that confirmed the NF1 gene mutation. None of the 14 patients underwent lung biopsy.

The chest CT scans evaluated were acquired in different scanners at various institutions. In all cases, the high-resolution technique was used and slices were obtained from the lung apices to the lung bases. Thin axial slices, 1-2 mm in thickness, were acquired with the patient in the supine position at end inspiration, a high-pass filter being used in order to reconstruct the images.

The images were reconstructed with a high-pass filter and a 512 × 512 matrix. The lung fields were evaluated with windows of 1,200-1,600 Hounsfield units (HU) in width and centered at a level between -500 HU and -700 HU. For the evaluation of the mediastinum, the window width was 350-450 HU and the window level was 10-20 HU. Among the 14 cases evaluated, intravenous iodinated contrast was used in only one.

The CT scans were analyzed by two chest radiologists, working independently, and disagreements were resolved by consensus. The radiologists were blinded to the demographic and clinical characteristics of the patients. The CT scans were evaluated for the presence and distribution of ground-glass opacities, reticulation, pulmonary cysts, bullae, and emphysema. The criteria for defining the findings were those listed in the Fleischner Society glossary of terms for thoracic imaging^**(12)**^. The terminology used is that established in the terminology consensuses issued by the Brazilian College of Radiology and Diagnostic Imaging^**(13)**^ and by the Imaging Department of the Brazilian Thoracic Association^**(14)**^. The presence of tracheobronchial neurofibromas and neurofibromas in the chest wall was also recorded, as was that of mediastinal and pleural lesions.

Pulmonary cysts were defined as rounded, circumscribed parenchymal air spaces, surrounded by an epithelial or fibrous wall, the thickness of which could be uniform or variable. Bullae were defined as air spaces (focal, rounded areas of lucency or reduced attenuation on CT) that were ≥ 1 cm diameter, delimited by thin (≤ 1 mm thick) walls. Pulmonary emphysema was defined as air spaces that were enlarged, distally from the terminal bronchioles, with destruction of the alveolar walls.

**Table 1:** Distribution of chest CT findings in patients with NF-DLD..

Finding	(N = 14)
Multiple cysts, n (%)	13 (92.9)
Emphysema, n (%)	8 (57.1)
Subpleural bullae, n (%)	6 (42.9)
Ground-glass opacities and reticulation, n (%)	1 (7.1)
Cutaneous or subcutaneous nodules, n (%)	12 (85.7)
Tracheobronchial nodules, n (%)	1 (7.1)

**Figure 1:**
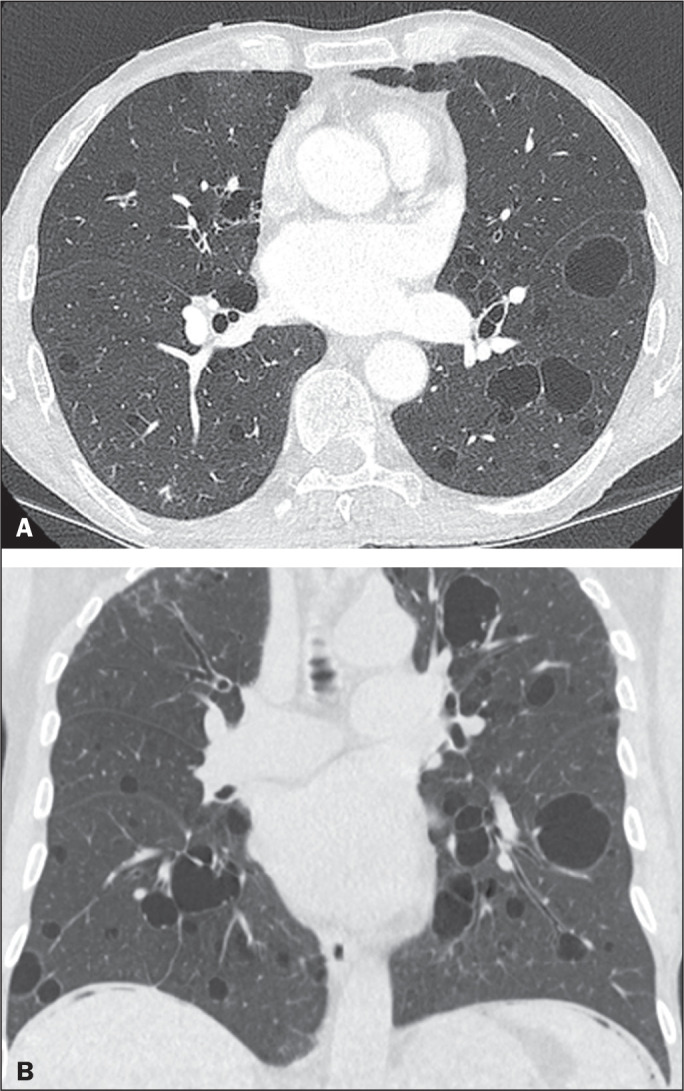
Chest CT of a 75-year-old man with NF1 who was a nonsmoker. Axial and coronal slices (A and B, respectively) showing multiple bilateral pulmonary cysts, some larger than 10 mm, with thin walls, predominantly in the lower lung fields, with a random distribution. Note the multiple cutaneous nodules in the chest wall.

**Figure 2:**
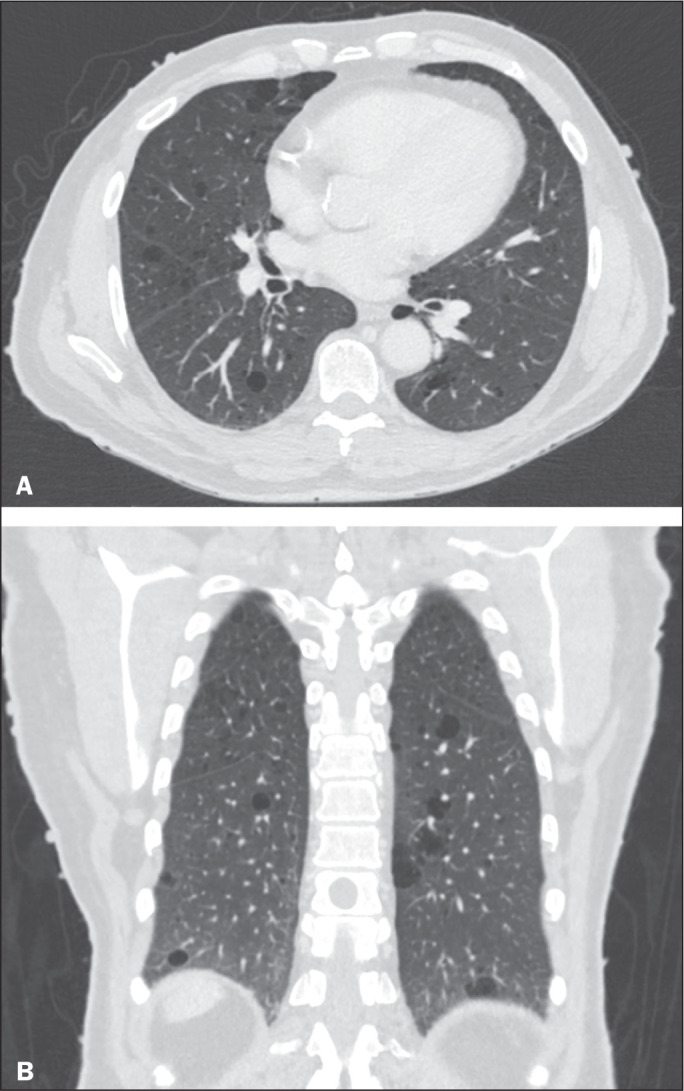
Chest CT of a 65-year-old man with NF1 and an unknown smoking history. Chest CT. Axial and coronal slices (A and B, respectively) showing multiple, bilateral, thin-walled cysts, smaller than 10 mm, predominantly in the lower lung fields, with a random distribution. Note the multiple cutaneous nodules in the chest wall.

**Figure 3:**
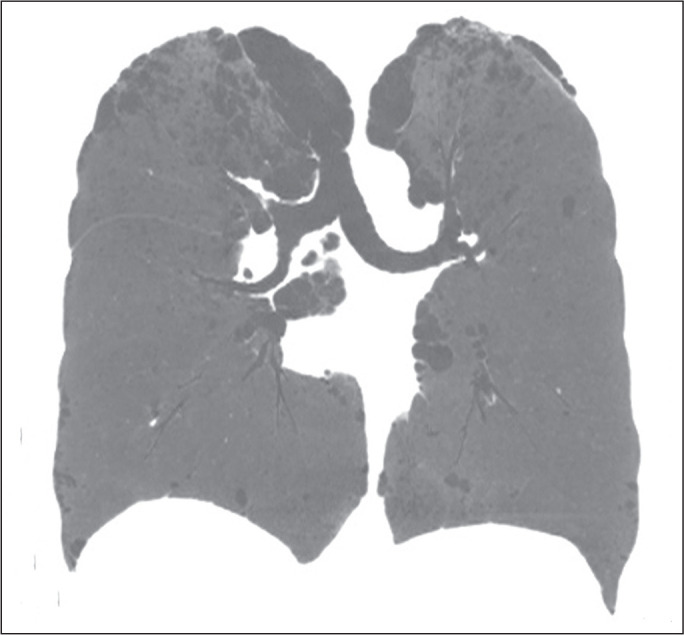
Chest CT of a 55-year-old woman with NF1 who was a nonsmoker. Coronal slice, with minimum intensity projection, showing bilateral centrilobular emphysema and subpleural bullae, predominantly in the upper lung fields .

**Figure 4:**
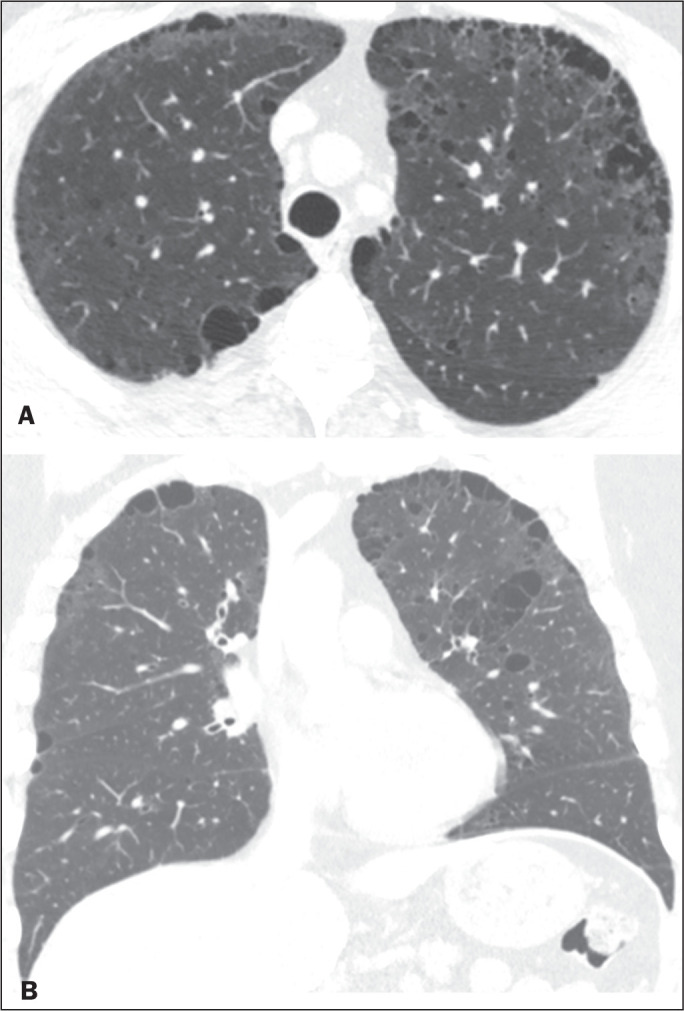
Chest CT of a 45-year-old man with NF1 who was a nonsmoker. Axial and coronal slices (A and B, respectively) showing small subpleural bullae and paraseptal emphysema, bilaterally and peripherally, in the upper lung fields, together with small thin-walled cysts in the lung parenchyma.

**Figure 5:**
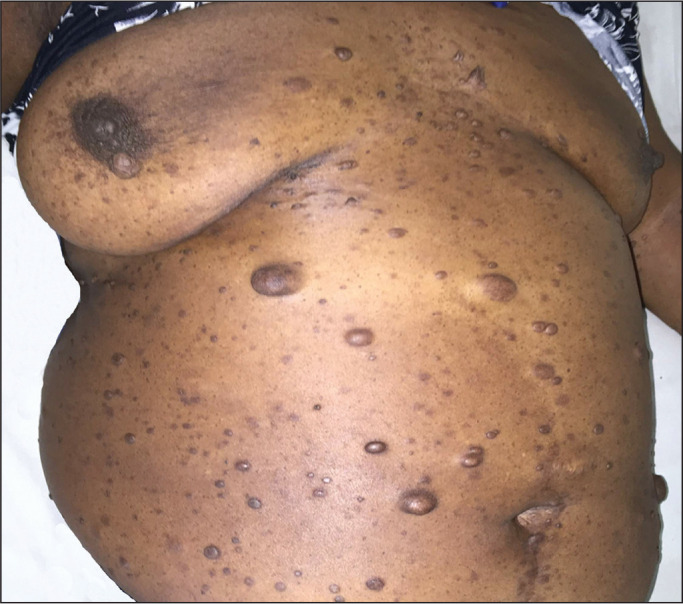
Photograph of a 48-year-old woman with NF1 and an unknown smoking history, showing multiple pedunculated cutaneous neurofibromas in the anterior and abdominal portions of the chest wall.

The changes were characterized in terms of laterality (bilateral or unilateral), as well as in terms of their distribution in the axial axis (central, peripheral, or random) and craniocaudal axis (upper, lower, or diffuse). Statistical data were calculated for each variable. Age is reported as median and range, whereas all other variables are reported as absolute and relative frequencies.

## RESULTS

### Clinical and epidemiological aspects

We evaluated 14 patients with NF1, of whom eight (57.1%) were women and six (42.9%) were men. The sample comprised 13 adults and one child. The median age was 55 years (range, 11-75 years). Of the 14 patients, eight (57.1%) were nonsmokers and three (21.4%) were smokers. For the remaining three patients (21.4%), data regarding smoking history were not available. Clinical symptom data were available for 11 (78.6%) of the patients. At the time of the chest CT, five (45.5%) of those patients had respiratory complaints and six (54.5%) were asymptomatic. The most common complaint was cough (in 100%), followed by dyspnea (in 80%) and pleuritic chest pain (in 20%). Of the five symptomatic patients, three (60%) were smokers. On physical examination, changes had been observed in eight of the patients: cutaneous or subcutaneous chest wall neurofibromas in seven (50%), café au lait spots in five (35.7%), Lisch nodules in two (14.3%), and axillary freckles in two (14.3%). On the basis of the clinical data available, we attempted to exclude all possible differential diagnoses of cystic lesions.

### Chest CT aspects

#### Chest CT patterns

In our study, the most common pulmonary finding on chest CT was that of multiple pulmonary cysts (Figures 1 and 2), which were reported in 13 (92.9%) of the patients, followed by emphysema (Figure 3), in eight (57.1%), and subpleural bullae (Figure 4), in six (42.9%). Ground-glass opacities and peripheral reticulations were reported in only one patient (7.1%). As can be seen in Table 1, cutaneous or subcutaneous neurofibromas in the chest wall (Figure 5) were found in 12 patients (85.7%) and tracheobronchial neurofibromas were found in one (7.1%). In the majority of cases, the three main findings (multiple cysts, emphysema, and subpleural bullae) were found in combination.

#### Distribution of chest CT changes

The pulmonary involvement was bilateral in 12 (85.7%) of the 14 cases evaluated and unilateral in two (14.3%). The distribution of the involvement was diffuse in four patients (28.6%), predominantly in the upper lung fields in eight (57.1%), and predominantly in the lower lung fields in two (14.3%). In the axial axis, the distribution was predominantly peripheral in one case (7.1%), central in two (14.3%), and random in 11 (78.6%).

Pulmonary cysts were found in 13 cases (92.9%), being bilateral in 11 (84.6%) of those cases and unilateral in two (15.4%). The distribution of the cysts was diffuse in four (30.8%) of those 13 patients, predominantly in the lower lung fields in two (15.4%), and predominantly in the upper lung fields in seven (53.9%). As for the distribution in the axial axis, multiple cysts were characterized as random in 10 (76.9%) of those 13 patients, central in two (15.4%), and peripheral in one (7.7%). Of the 13 patients with pulmonary cysts, seven (53.8%) were nonsmokers. All three of the smoking patients had pulmonary cysts.

Pulmonary emphysema was identified in eight (57.1%) of the 14 cases evaluated, being bilateral with a predominance in the upper lung fields in all cases. The emphysema was classified as centrilobular in two (25.0%) of the eight cases and paraseptal in three (37.5%), whereas it was centrilobular and paraseptal in three (37.5%). Although emphysematous changes were seen in all three of the smokers in our sample, they were also seen in two (25%) of the eight nonsmokers.

Bullae were found in six (42.9%) of the 14 cases evaluated. In all six cases, the bullae were bilateral, peripheral (subpleural), and predominantly located in the upper lung fields.

Ground-glass opacities and reticulation were found in only one (7.1%) of the 14 patients studied. The observed changes were bilateral, with a peripheral distribution and no evident predominance in the craniocaudal axis. That patient was a smoker.

## DISCUSSION

In the present study, there was a slight predominance of women, who accounted for 57.1% of the sample, the male:female ratio being 1:1.3. That is consistent with the findings of other studies of NF-DLD, in which no predilection for sex has been reported^**(15-18)**^. The age at diagnosis reported in the literature ranges from 16 to 72 years^**(19)**^, which is comparable to the age range of 11 to 75 years observed in our study sample. It is known that pulmonary involvement develops later in the course of NF-DLD, typically in the third or fourth decade of life^**(20)**^. Our sample of patients with NF-DLD included nonsmokers and smokers. The literature reviewed suggests that NF1 can evolve to NF-DLD in smokers and nonsmokers, confirming that such pulmonary involvement is independent of smoking status.

In our study sample, the main chest CT finding was multiple pulmonary cysts, which were observed in 13 patients (92.9%), followed by pulmonary emphysema, in eight (57.1%), subpleural bullae, in six (42.9%), ground-glass opacities/reticulation, in one (7.1%), and tracheobronchial nodules, in one (7.1%). Regarding the distribution of the pulmonary lesions in the two axes, it was bilateral in 12 patients (85.7%), predominantly in the upper lung fields in eight (57.1%), and random in 11 (78.6%). The detailed evaluation of the three main imaging findings (pulmonary cysts, pulmonary emphysema, and subpleural bullae) revealed the following: the pulmonary cysts were bilateral in 11 (84.6%) of the 13 patients affected, were located predominantly in the upper lung fields in seven (53.8%), and had a random distribution in 10 (76.9%); the pulmonary emphysema was bilateral and predominantly in the upper lung fields in all patients, the morphology being centrilobular in two (25.0%) of the eight cases and paraseptal in three (37.5%), whereas it was centrilobular and paraseptal in three (37.5%); and the distribution of the subpleural bullae, in all cases, was bilateral, peripheral, and predominantly in the upper lung fields.

Recent studies in the radiology literature of Brazil have highlighted the importance of imaging methods in the assessment of the chest^**(21-26)**^. However, there have been few studies describing the pulmonary CT findings in patients with NF1. In our search of the literature, we found only one such study, a case series conducted by Ueda et al.^**(19)**^, who detailed the specific prevalence of each chest CT finding in a sample of 88 patients diagnosed with NF1, the main findings being subcutaneous nodules (in 51%), cutaneous nodules (in 39%), scoliosis (in 23%), emphysema (in 18%), pulmonary cysts (in 15%), mediastinal masses (in 15%), and ground-glass nodules (in 9%). In that study, the pulmonary cysts were in the upper lung fields in 85% of cases, in the middle lung fields in 46%, and in the lower lung fields in 54%. Peripheral cysts were found in all cases, central cysts being seen only 31%. Regarding emphysema, there was a statistically significant predominance of peripheral distribution in the upper lung fields, as was observed in 94% of the patients. The discrepancies between our findings and those of Ueda et al.^**(19)**^, in terms of the prevalence of pulmonary changes, is due to the fact that those authors did not limit their sample to patients with NF-DLD exclusively, including all patients with NF1, which lowered the proportions of patients with a given chest CT finding. Nevertheless, their finding that the majority of the lesions were in the upper lung fields is in agreement with our findings.

In addition to the morphology of lung injuries, Ueda et al.^**(19)**^ assessed the relationship between smoking and the frequency of pulmonary findings in NF-DLD. The authors concluded that there was no significant difference between the patients who were smokers and those who were nonsmokers in terms of the prevalence of pulmonary cysts (5.3% and 7.1%, respectively), suggesting that smoking has no effect on the occurrence or morphological aspect of such cysts in patients with NF1^**(19)**^. In contrast, their results suggest that pulmonary emphysema is strongly affected by smoking status in patients with NF1, the prevalence of emphysema being significantly higher among the smokers than among the nonsmokers (63.2% vs. 4.8%).

The most important finding of the present study was the high (92.9%) prevalence of multiple pulmonary cysts, which were seen in all but one of the cases evaluated. In addition, our results suggest that there is no significant difference between smokers and nonsmokers in terms of the prevalence of such cysts. However, we found that the prevalence of pulmonary emphysema was higher in the patients who were smokers than in those who were nonsmokers (100% vs. 25%), which suggests that emphysema cannot be attributed to NF1 alone; smoking status must be taken into consideration. It is noteworthy that only one of the patients in our sample presented with ground glass opacities/reticulation on chest CT, and that that patient was a smoker. In previous studies, interstitial infiltrates on chest CT have been described in patients with NF-DLD, although other potential causes of the infiltrates, such as smoking, have also been observed in those patients^**(6,19)**^.

Cysts and bullae have been detected in smokers and former smokers, leading some to question the validity of their association with NF1^**(6,27)**^. However, there is growing evidence of pulmonary involvement in nonsmoking individuals with NF1. Zamora et al.^**(28)**^ found that, of 16 patients with NF-DPD for whom smoking histories were available, four (25%) were nonsmokers. Oikonomou et al.^**(29)**^ evaluated six never-smokers with NF1 (age range, 23-61 years) who underwent chest CT to investigate the possibility of NF-DLD. Those authors found evidence of cystic lung disease in all six patients. The cysts were predominantly in the upper lung fields, were shown to have “extremely thin, barely perceptible, but well-defined walls”, and ranged from 2 mm to 18 mm in diameter. Five of the patients had fewer than 20 cysts, whereas more than 100 cysts were identified in the remaining patient^**(29)**^.

Our study had some limitations. Because it was a cross-sectional study, it was not possible to evaluate the evolution of the changes in the lung parenchyma. In addition, the retrospective design and small size of the patient sample limited our analysis of the clinical data and smoking history. Despite those limitations, we believe that our findings are relevant, because NF1 is a rare disease and pulmonary involvement in NF1 is even rarer. To our knowledge, there has been only one study evaluating CT patterns in a sample of patients with NF-DLD larger than our sample^**(19)**^.

In conclusion, NF-DLD appears to manifest essentially as three patterns on chest CT, most commonly as multiple pulmonary cysts, followed by pulmonary emphysema and bullae. In most of the patients in our sample, the changes were bilateral, were located in the upper lung fields, and had a random distribution. Pulmonary cysts can be seen in smoking and nonsmoking patients alike. Although pulmonary emphysema can be seen in nonsmokers with NF1, there is insufficient evidence to conclude that it is due solely to NF1.
